# A Multi-Biomarker Approach to Increase the Accuracy of Diagnosis and Management of Coronary Artery Disease

**DOI:** 10.3390/jcdd11090258

**Published:** 2024-08-23

**Authors:** Lenka Hostačná, Jana Mašlanková, Dominik Pella, Beáta Hubková, Mária Mareková, Daniel Pella

**Affiliations:** 1 Department of Clinical Biochemistry, Medirex, a.s., Magnezitárska 2/C, 040 13 Košice, Slovakia; loravcov@gmail.com; 2 Department of Medical and Clinical Biochemistry, Faculty of Medicine, Pavol Jozef Šafárik University in Košice, Trieda SNP 1, 040 11 Košice, Slovakia; beata.hubkova@upjs.sk (B.H.); maria.marekova@upjs.sk (M.M.); 3 1st Department of Cardiology of the East Slovak Institute of Cardiovascular Diseases, Faculty of Medicine, Pavol Jozef Šafárik University in Košice, Trieda SNP 1, 040 11 Košice, Slovakia; dominik.pella@upjs.sk; 4 2nd Department of Cardiology of the East Slovak Institute of Cardiovascular Diseases, Faculty of Medicine, Pavol Jozef Šafárik University in Košice, Trieda SNP 1, 040 11 Košice, Slovakia

**Keywords:** coronary artery disease, coronary angiography, inflammatory biomarkers

## Abstract

Non-invasive possibilities of predicting cardiovascular risk and monitoring the treatment and progression of coronary artery disease (CAD) are important subjects of cardiovascular research. Various inflammatory markers have been identified as potential biomarkers of CAD, including interleukin-6 (IL-6), lipocalin-2 (LCN-2), growth differentiation factor 15 (GDF-15), and T cell immunoglobulin and mucin domain-3 (TIM-3). This research aims to identify their utility in the investigation of CAD severity and progression. The basic anthropometric parameters, as well as the levels of urea, creatinine, CRP, leukocytes, fibrinogen, and biomarkers of inflammation, were measured in 130 patients who underwent coronary angiography. In male patients, divided according to findings on coronary angiography, we observed an increasing expression of GDF-15 with increasing stenosis (with worsening findings). In females, we observed increasing fibrinogen expression with increasing stenosis, i.e., findings on coronary angiography. Correlation analysis did not confirm the relationship between TIM-3, LCN and 2, IL-6 and the severity of findings obtained by coronary angiography; however, the correlation of TIM-3 and LCN-2 expression was positive with the finding, and the correlation of IL-6 with the finding was surprisingly negative. Understanding the role of these inflammatory markers in CAD can be helpful in risk stratification, guiding therapeutic strategies, and monitoring treatment responses in patients with CAD.

## 1. Introduction

Atherosclerotic cardiovascular diseases (ASCVDs) are the most common chronic vascular disease and a major public health problem worldwide [1]. ASCVDs continue to be the leading cause of mortality worldwide, and coronary artery disease (CAD) is the most prevalent form [2,3]. According to the latest WHO data, 10,654 people die of CAD in Slovakia annually, which represents about 23.8% of the total number of deaths [4].

The main cause of coronary artery disease, atherosclerosis, was traditionally regarded as a disease of cholesterol accumulation caused by the retention of lipoproteins, including low-density lipoprotein (LDL), in the intima of coronary arteries. Still, this hypothesis was followed by a newer inflammatory hypothesis, where the inflammatory process plays a significant role in the development and progression of CAD [5].

Various inflammatory markers have been identified as potential inflammation indicators and cardiovascular risk predictors. Understanding the role of these inflammatory markers in CAD can help in risk stratification, guiding therapeutic strategies, and monitoring treatment response in patients with CAD [6]. Targeting inflammatory pathways and reducing inflammation may have therapeutic benefits in preventing and treating CAD.

Lipocalin-2 (LCN-2) triggers the inflammatory process in human atherosclerotic tissue directly [7]. LCN-2 is a 25 kDa protein secreted by activated neutrophils, adipocytes, and macrophages [8]. LCN-2 is an acute-phase protein that can act as a pro-inflammatory and anti-inflammatory mediator, depending on the context. LCN-2 can induce the production of pro-inflammatory cytokines, such as interleukin-6 (IL-6) and tumor necrosis factor-alpha (TNF-alpha), by immune cells, further amplifying the inflammatory response [7,8]. LCN-2 facilitates the adhesion and migration of immune cells to sites of inflammation, promoting tissue infiltration and inflammation. On the other hand, it also has an anti-inflammatory role. LCN-2 is involved in the sequestration and transport of iron, which is essential for immune function. It can modulate the inflammatory response and may play a role in resolving inflammation and promoting tissue repair by modulating immune cell function and cytokine production [9]. The elevated levels of LCN-2 have been associated with various inflammatory diseases, including cardiovascular diseases, chronic kidney disease, inflammatory bowel disease, and sepsis [10].

IL-6 is an acute-phase protein that plays a significant role in the inflammatory response, vascular inflammation, and development of CAD. IL-6 is expressed mainly by activated macrophages but also by endothelial cells and smooth muscle cells [11]. By increasing metalloproteinase gene expression, it contributes to the remodeling of connective tissue, and focal overexpression of activated metalloproteinases can promote destabilization and degradation of the plaque’s fibrous cap [12].

The relationship between IL-6 and CAD is bidirectional. The atherosclerotic process that causes subsequent CAD is associated with increased IL-6 production due to inflammation, and on the other hand, increased IL-6 values, determined by extrinsic factors such as obesity, social stress, smoking, and pollution, may increase the atherosclerotic risk [3].

Other promising mortality and disease progression predictors in patients with atherosclerosis and CAD are growth differentiation factor 15 (GDF-15), T-cell immunoglobulin, and mucin domain-3 (TIM-3).

GDF-15 represents a stress-responsive member of the transforming growth factor-β (TGF-β) cytokine superfamily. GDF-15 has anti-inflammatory properties and can attenuate inflammation by inhibiting pro-inflammatory cytokine production and modulating immune cell function. Moreover, GDF-15 reduces the increase in IL-6, TNF-α, and IL-1β expression in serum and liver tissue and inhibits the activation of the IκBα/NF-κB pathway by disrupting TGFβ-activated kinase 1 (TAK1) phosphorylation in Kupffer cells [13]. GDF-15 is released in high concentrations in response to mitochondrial dysfunction, oxidative stress, or metabolic stress and/or through stimulation by pro-inflammatory cytokines [14].

TIM-3 is expressed on various immune cells, including T cells, macrophages, and dendritic cells, and modulates inflammatory responses. It is involved in the control of T-cell exhaustion and immune checkpoint regulation [15]. Dysregulated T-cell responses have been implicated in the pathogenesis of CAD, where T-cells contribute to vascular inflammation, endothelial dysfunction, and plaque destabilization. TIM-3 may influence the balance between pro-inflammatory and anti-inflammatory T-cell responses in CAD [15].

GDF-15 and TIM-3 play important roles in modulating immune responses and have been implicated in various physiological and pathological processes, including inflammation, autoimmunity, cancer, and cardiovascular diseases [16,17].

The combination of Growth Differentiation Factor-15 (GDF-15)**,** Interleukin-6 (IL-6)**,** Lipocalin-2 (LCN-2), and T-cell immunoglobulin and mucin-domain containing-3 (TIM-3) provide a multi-faceted approach to the diagnosis and understanding of coronary artery disease (CAD), as each biomarker reflects distinct biological processes involved in the disease. Together, these markers offer insights into inflammation, immune responses, tissue remodeling, and vascular dysfunction that contribute to atherosclerosis and CAD progression. Each of these biomarkers reflects a different aspect of the disease process in CAD, and their combined measurement can enhance the accuracy of diagnosing early-stage CAD, assessing disease severity, and predicting outcomes.

Elevated levels of IL-6 and TIM-3 indicate active inflammation and immune dysregulation, which are central to atherosclerotic plaque formation and instability [11,15]. The elevation of GDF-15 and LCN-2 in CAD patients suggests that there is ongoing vascular injury, contributing to plaque formation and arterial stiffening [10,18]. High levels of IL-6, GDF-15, and LCN-2 are associated with plaque instability, which increases the risk of rupture and acute coronary events [8,11,12,13]. This multi-biomarker approach could increase the accuracy of diagnosing and managing coronary artery disease. Diagnosing early-stage coronary artery disease (CAD) is challenging because it often does not present with clear symptoms.

## 2. Materials and Methods

The examined sample consisted of patients (*n* = 130) who were admitted to the 1st Department of Cardiology of the East Slovak Institute of Cardiovascular Diseases, a.s., the clinical educational base of the Faculty of Medicine UPJŠ in Košice, in suspicion of CAD. All patients were examined by coronary angiography and divided according to the results into two groups—a control group and a group with pathological results. The control group (*n* = 34) consisted of patients with physiological results made by 16 women (48.5%) and 18 men (51.5%) with a mean age of 61.5 years. The second group consisted of 96 patients with pathological results documented by selective coronary angiography findings 1–5 (Table 1). In the second group, the number of women with pathological results was 29 (30.2%) and the number of men was 67 (69.8%). The mean age in the second group was 65 years. According to the pathological results of selective coronary angiography, patients were categorized into 5 groups. In the group with finding 1 (CAD-RADS 1), there were 4 women and 5 men, with finding 2 (CAD-RADS 2)—5 women and 6 men, with finding 3 (CAD-RADS 3)—10 women and 14 men, with finding 4 (CAD-RADS 4)—5 women and 27 men, and with finding 5 (CAD-RADS 5)—5 women and 15 men.

The group of patients did not include patients with already known ischemic heart disease and myocardial infarction (verified by selective coronary angiography), patients after a stroke, and at the same time we focused on patients of productive age.

Blood samples were collected using BD Vacutainer^®^ EDTA test tubes (Eysins, Switzerland) and analyzed by the ELISA method with the following assay kits according to the manufacturer’s instructions: Lipocalin 2 (Human Lipocalin 2 ELISA kit (NGAL), ab113326, Abcam, Cambridge, MA, USA) detection method: colorimetric, assay type: sandwich (quantitative), range: 4.1–1000 pg/mL, sensitivity < 4 pg/mL; TIM-3 (Human TIM-3 ELISA kit, ab231932, Abcam, Cambridge, MA, USA) detection method: colorimetric, assay type: sandwich (quantitative), range: 78.13–5000 pg/mL, sensitivity 14.6 pg/mL; IL-6 (Human IL-6 ELISA kit, ab46027, Abcam, Cambridge, MA, USA) detection method: colorimetric, assay type: sandwich (quantitative), range: 6.25–200 pg/mL, sensitivity < 2 pg/mL; GDF-15 (Human GDF-15 ELISA kit, ab155432, Abcam, Cambridge, MA, USA) detection method: colorimetric, assay type: sandwich (quantitative), range: 1.1–800 pg/mL, sensitivity < 2 pg/mL.

All statistical analyses were performed using IBM SPSS Statistics 29.0.1.0 (IBM, Armonk, New York, NY, USA). Testing for Normality was performed by the Kolmogorov–Smirnov test. Differences between group means/medians were calculated using a two-sample *t*-test, assuming or not assuming equal variances (based on Levene’s Test for Equality of Variances). The strength of the linear relationship between the two variables was expressed by the Pearson correlation coefficient; a *p*-value < 0.05 was assumed to be statistically significant. Means and standard deviations are reported in terms of the original distributions, while parameters with lognormal distribution are expressed as median with interquartile range. The research was approved on 24 January 2019, by the Ethics Committee of UPJŠ LF -18N/2021.

## 3. Results

The study included 130 patients admitted with suspected CAD. Based on the coronary angiography examination, the patients were classified according to the severity of the findings. Their basic anthropometric parameters, as well as the levels of urea, creatinine, CRP, leukocytes, fibrinogen, and biomarkers of inflammation: LCN-2, GDF-15, IL-6, TIM-3, concerning the findings of coronary angiography, are shown in Table 2.

Correlation analysis confirmed the statistical significance between coronary angiography findings and gender (Pearson correlation factor −0.225, *p* < 0.01 **) and coronary angiography findings and blood pressure (Pearson correlation factor −0.186, *p* < 0.035 *) in the group of patients, which we divided according to the findings on coronary angiography.

Correlation analysis did not confirm the relationship between selected pro- and anti-inflammatory biomarkers and the severity of findings obtained by coronary angiography; however, the correlation of GDF-15, TIM-3, and LCN-2 expression was positive with the finding, and the correlation of IL-6 with the finding was surprisingly negative (Table 3).

The severity of the findings obtained by coronary angiography was worse in men; most of them had severe non-obstructive CAD (RADS 4) (31.8%), while up to 35.6% of women had no detected stenosis (RADS 0) (Table 4).

Since Pearson’s correlation showed statistically significant differences between findings and blood pressure, we also focused on the blood pressure values depending on individual findings. In all groups of patients, blood pressure was higher than normal, i.e., 136.875 (RADS 3), which according to standards is the stage of hypertension. Surprisingly, the highest blood pressure values (average 146.618) were measured in the group of patients with RADS 0 (Table 5).

Since Pearson’s correlation showed statistically significant differences between findings and gender, we divided the patients into groups according to the coronary angiography findings into men and women (Table 6). After this division, we noticed a statistically significant correlation in men, where the finding is negatively correlated with blood pressure (Pearson correlation factor −0.238, *p* < 0.028 *) and positively with GDF-15 expression (Pearson correlation factor −0.271, *p* < 0.013 *), and in women, the finding is positively correlated with the level of fibrinogen (Pearson correlation factor −0.294, *p* < 0.05 *) (Table 6). We observe an opposite trend between men and women for some parameters. The severity of the finding in men decreases with increasing age, urea, and the number of leukocytes, while the trend is the opposite for women.

In addition to blood pressure, significant correlations emerged with GDF-15 expression, but only in men. GDF-15 in women was not significantly correlated with the findings; the correlation was even negative. We divided the male patients according to the findings and observed an increasing expression of GDF-15 with increasing stenosis (with a worsening finding) (Table 7).

We divided the female patients according to the findings from the coronary angiography and used descriptive statistics to compare the fibrinogen expression between the patients in individual groups. We observed an increasing expression of fibrinogen with increasing stenosis, i.e., a finding on coronary angiography (Table 8). We noted an exception in the RADS 1 group, but this group also had the fewest patients.

**Figure 1:**
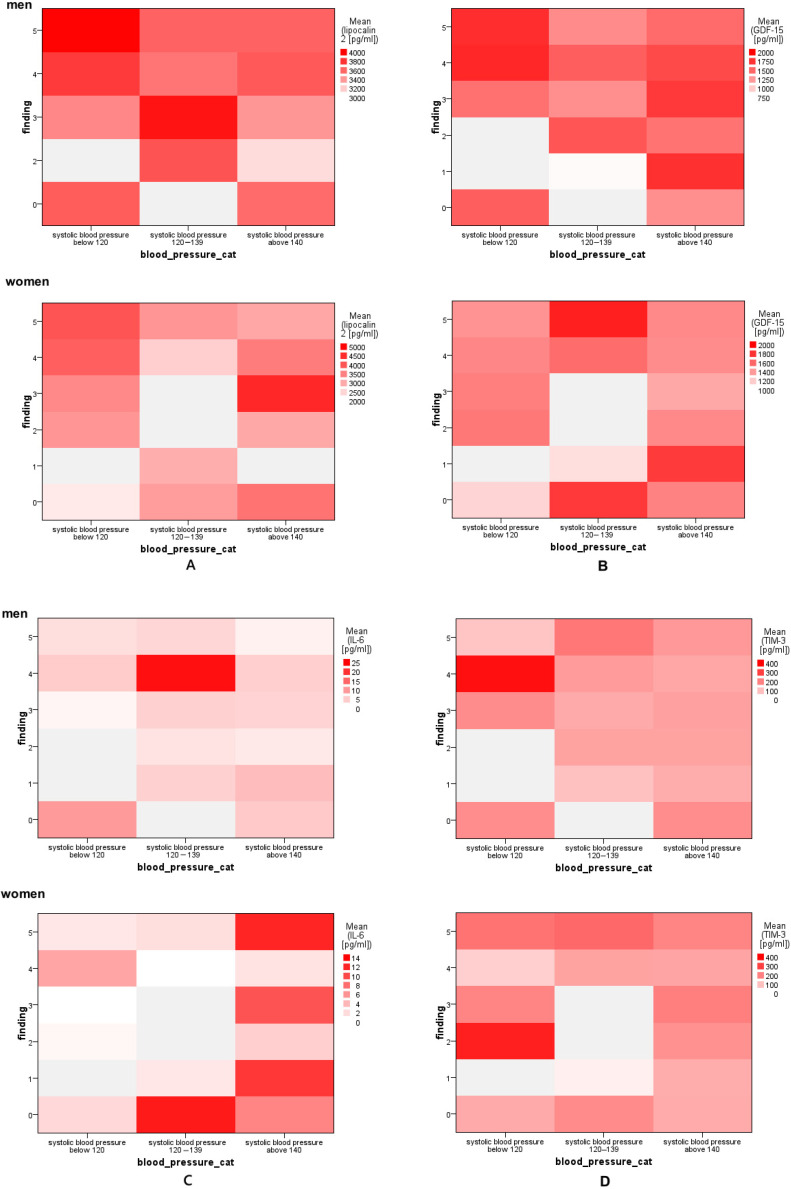
Heat maps as a correlation of 3 parameters: finding, blood pressure, and (**A**): LCN-2, (**B**): GDF-15, (**C**): IL-6, and (**D**): TIM-3.

Since the Pearson correlation test showed a significant correlation between the blood pressure of the patients and also between the sexes, and we mainly focused on the determination of pro-inflammatory and anti-inflammatory biomarkers, we chose the method of heat maps, which is suitable for the correlation of three parameters, in which the size of individual values within the data set is displayed as a color shade (Figure 1).

Correlation matrix heat map (correlogram) (blood pressure–finding LCN-2): In both men and women with normal systolic blood pressure (below 120 mmHg), LCN-2 rises with the severity of the finding. This correlation is not confirmed in women with BP above 120 mmHg, nor in men with BP in ranges from 120 to 139 mmHg. On the contrary, in men with BP above 140 mmHg, the concentration of LCN-2 increases again with the severity of the finding.

Correlogram (blood pressure–finding GDF-15): The correlation of GDF-15 with the severity of the finding depending on the systolic blood pressure is very different in men and women. While in men with pressure up to 120 mmHg, GDF-15 increases with severity, the opposite is true in women. There is a strong direct correlation between GDF-15 and the severity of the finding in women with pressure in the range of 120 to 139 mmHg; the correlation was not confirmed in men. In women with a pressure above 140 mmHg, the correlation between GDF-15 and the severity of the finding is negative, while in men with a pressure above 140 mmHg, the GDF-15 values are high regardless of the severity of the finding.

Correlogram (blood pressure–finding IL-6): In men, the highest value of IL-6 was recorded in patients with BP between 120 and 139 mmHg with RADS-4 finding severity. In women with similar pressure, a high level of IL-6 was observed in patients without serious findings, while high levels were also measured in patients with pressure above 140 mmHg without significant correlation.

Correlogram (blood pressure–finding TIM-3): In both female and male patients, we noted a correlation between the levels of TIM-3 and the severity of the finding in the case that they had a pressure in the range of 120 to 139 mmHg. In patients with physiological pressure (up to 120 mmHg), this correlation was positive in men and negative in women. In patients with pressure above 140 mmHg, regardless of gender, TIM-3 levels were average and did not show any correlation with the severity of the finding.

## 4. Discussion

Selective coronary angiography has become the gold standard for CAD diagnosis, which can be used to monitor coronary artery stenosis and the degree of lesions. This examination, however, requires hospitalization and may lead to related complications. Despite the promising results of the use of selective coronary angiography, research into CAD is constantly trying to find new, less burdensome, and less interventional coronary artery imaging techniques (e.g., CT coronary angiography, optical coherence tomography), which, together with the determination of prospective biomarkers in the patient’s biological fluids, could expand the non-invasive possibilities of predicting the risk of occurrence while also monitoring the treatment and progression of these diseases.

The association between many pro-inflammatory and anti-inflammatory markers and the progression of CAD has been demonstrated by many studies [8,11,19,20,21,22]. In our study, we focused on investigating four biomarkers in patients with suspicion of CAD, divided according to the results of coronary angiography. The basic anthropometric parameters, as well as the levels of urea, creatinine, CRP, leukocytes, fibrinogen, and biomarkers of inflammation, were measured.

Statistical significance was found between men and women, who were divided according to coronary angiography findings and also coronary angiography findings and blood pressure. Up to 31.8% of men had a stenosis of 70–99%. No stenosis was noted in the largest percentage of women, up to 35.6%. Similar results were also obtained in the study by Sayed et al., where they recorded 20.1% of women and 79.9% of men with CAD [23].

CAD tends to develop approximately 7–10 years later in women compared to men [24]. This is thought to be due to the protective effects of estrogens on premenopausal women. However, post-menopause, the risk increases significantly. Kim et al. (2024) in their study, described that the majority of patients with CAD up to the age of 50 were men. However, this trend reversed when patients were between 60 and 69 years of age, or aged 80 years or older; 72.2% of cases were found in women and 27.8% in men [25]. Another very important point is that traditional diagnostic tests such as stress tests or nuclear imaging may be less sensitive in women due to differences in chest size, breast tissue, and hormonal influences [26]. Male–female differences in CAD involve various aspects, including risk factors, pathophysiology, clinical presentation, diagnostic challenges, treatment responses, and outcomes. These differences highlight the need for gender-specific approaches to the prevention, diagnosis, and treatment of CAD to improve outcomes for both men and women.

Significant correlations emerged with GDF-15 expression, but only in men. We observed an increasing expression of GDF-15 with increasing stenosis (with a worsening finding). Increased levels of GDF-15 have been established as a predictive factor for several cardiovascular diseases, including CAD, acute coronary syndromes (ACS), and chronic heart failure, as well as for all-cause cardiovascular mortality. Study Wang et al. (2017) evaluated the incremental prognostic value of GDF-15, which provided more information than other biomarkers. GDF-15 was significantly associated with stable CAD and ACS [27]. Data from the Framingham Heart study, in which 85 biomarkers were evaluated, showed that GDF-15 was the only marker with a significant association with the three outcome results: atherosclerotic events, heart failure, and mortality [28].

In women, we observed an increasing expression of fibrinogen with increasing stenosis, i.e., a finding on coronary angiography, even though the mean fibrinogen level was at the upper limit of physiological values (1.8–3.5 g/L). Fibrinogen exceeded this limit only in female patients by finding 4 (stenosis of 70–99%). In the study by Surma et al. (2022), it was described that people whose fibrinogen plasma concentration was in the second and third terciles (concentrations: 2.7–3.1 g/L and 3.1–7.0 g/L, respectively) were characterized by a significantly higher frequency of CAD than those in the first tercile (1.3–2.7 g/L) [29]. In the study by Peng et al. (2016), cumulative survival curves showed that the risk of all-cause mortality was significantly higher in subjects with plasma fibrinogen concentrations ≥ 3.17 g/L vs. those with <3.17 g/L (mortality rate, 11.5% vs. 5.7%, *p* < 0.001); and cardiac mortality rate—5.9% vs. 3.6%, *p* = 0.002). We determined fibrinogen values higher than 3 g/L in all our patients, both women and men [30]. A large study by Safdar et al. (2018), which included 2690 patients who underwent coronary angiography for ACS, confirmed that women younger than 55 years have a five times higher risk of myocardial infarction with non-obstructive coronary arteries (MINOCA) compared to men of the same age. These observations point to a different role for fibrinogen in women with CAD [31]. Thus, monitoring fibrinogen levels (mainly in females) may be valuable in risk stratification, prognosis, and treatment decisions to reduce the risk of adverse cardiovascular events.

In the case of atherosclerosis, numerous studies have reported that increased levels of serum LCN-2 correlate with atherosclerosis risk factors, disease severity burden, and mortality [32]. In a study by Zografos et al. (2009), LCN-2 concentrations were measured in 73 patients who underwent first-time coronary angiography for suspected CAD, and their associations with angiographic indexes of the severity of CAD (i.e., the number of diseased vessels and modified Gensini score) were estimated. Median serum LCN-2 levels in patients with angiographically confirmed CAD were significantly higher than those in patients with normal coronary arteries. Statistically significant correlations were observed between serum LCN-2 level, and the number of diseased vessels, and the modified Gensini score [33]. In our study, these findings were not proven. Correlation matrix heat map (correlogram) pressure–finding LCN-2, revealed that LCN-2 increases with severity in both men and women only with normal systolic blood pressure (below 120 mmHg). However, in our group of 130 patients, only 21 patients (15 men and 6 women) had normal systolic pressure. Many studies describe that LCN2 has a dual role in inflammation. Our LCN2 expression results do not point to either a pro-inflammatory or an anti-inflammatory role for this marker.

In the meta-analysis, which included eleven epidemiological studies, IL-6 emerged as a predictive factor for CAD. IL-6 baseline levels were associated with an increased risk of developing CAD at the end of the follow-up period [34]. In our study, IL-6 levels do not correlate with CAD progression, and it seems IL-6 has minimal or no direct pro-inflammatory or anti-inflammatory effects in specific contexts within coronary artery disease. In some cases, IL-6 may focus on cell survival, metabolic regulation, or tissue remodeling, which might not involve direct inflammatory processes.

TIM-3 is an immune checkpoint receptor expressed on various immune cells, including T cells, macrophages, and dendritic cells. By reducing pro-inflammatory proteins, TIM-3 may help control the chronic inflammation responsible for coronary artery disease progression. Xiao et al. ’s study described that the TIM-3 expression levels in the circulating monocytes of CAD patients were lower than those of the non-CAD group [16].

Du et al. reported that TIM-3 negatively regulates the production of reactive oxygen species and the secretion of pro-inflammatory cytokines in macrophages, and lipid deposition was observed after TIM-3 knockout [35]. Our results of TIM-3 expression have a different tendency in men and women, while in men, TIM-3 expression increases when going from patients without CAD to patients with minimal non-obstructive CAD and further increases in patients with mild non-obstructive CAD. In women, TIM-3 levels are higher in patients without CAD and decrease in patients with minimal non-obstructive CAD. However, significance was not confirmed between any patient groups. The correlogram (blood pressure–finding TIM-3) reveals a correlation between the levels of TIM-3 and the severity of the finding in the case that they had a pressure in the range of 120 to 139 mmHg in both men and women. Determination of the combination of GDF-15, IL-6, LCN-2, and TIM-3 markers can indeed complement coronary angiography in the diagnosis, risk assessment, and management of coronary artery disease (CAD). While coronary angiography provides direct imaging of coronary arteries to detect blockages or stenosis, measuring these biomarkers adds valuable information about underlying biological processes such as inflammation, immune regulation, and plaque stability that angiography alone cannot fully reveal.

## 5. Conclusions

While coronary angiography remains the gold standard for directly visualizing coronary artery blockages, these blood biomarkers can provide valuable information about the risk and presence of CAD, as well as the likelihood of future cardiovascular events. They can be used to guide clinical decision-making, especially in determining which patients might benefit from more invasive testing or aggressive management. Integrating these biomarkers into clinical practice can enhance the non-invasive assessment of CAD, potentially reducing the need for selective coronary angiography in certain cases.

Further research is needed to elucidate the precise mechanisms underlying the interplay between inflammation and CAD and to explore the potential therapeutic implications of targeting inflammatory pathways in the prevention and treatment of CAD.

## Figures and Tables

**Table 1:** Patient categorization according to coronagraphy angiography findings.

Category	Maximum Stenosis	Interpretation
CAD-RADS 0	0%	CAD absence
CAD-RADS 1	1–24%	Minimal non-obstructive CAD
CAD-RADS 2	25–49%	Mild non-obstructive CAD
CAD-RADS 3	50–69%	Moderate stenosis
CAD-RADS 4	70–99%	Severe stenosis
CAD-RADS 5	100%	Total coronary occlusion

**Table 2:** The basic anthropometric parameters, as well as the levels of urea, creatinine, CRP, leukocytes, fibrinogen, and biomarkers of inflammation: LCN-2, GDF-15, IL-6, TIM-3 concerning the findings by coronary angiography.

	RADS 0		RADS 1		RADS 2		RADS 3		RADS 4		RADS 5	
	Men, *n* = 18	Women, *n* = 16	Men, *n* = 5	Women, *n* = 4	Men, *n* = 6	Women, *n* = 5	Men, *n* = 14	Women, *n* = 10	Men, *n* = 27	Women, *n* = 5	Men, *n* = 15	Women, *n* = 5
age/years	62 ± 11	62 ± 11	63 ± 9	72 ± 12	68 ± 8	68 ± 13	65 ± 9	69 ± 5 *	64 ± 10	70 ± 5 *	61 ± 9	67 ± 11
BMI	30.69 ± 5.47	34.06 ± 11.4	28.08 ± 3.19	38.75 ± 5.46	30.97 ± 4.36	33.18 ± 9.56	31.76 ± 4.85	31.19 ± 4.99	31.48 ± 4.98	29.88 ± 8.70	29.81 ± 4.79	30.98 ± 3.58
BP *	148.89 ± 13.4	144.06 ± 17.34	138 ± 8.37	145 ± 18.71	145.83 ± 12.01	147 ± 31.54	134.64 ± 19.66 *	140 ± 11.55	138.98 ± 19.9	134.5 ± 11.24	137.00 ± 14.12 *	147 ± 26.83
Urea [mmol/L]	5.85 ± 1.42	5.54 ± 1.79	5.61 ± 1.21	6.37 ± 1.27	5.85 ± 0.75	5.29 ± 0.72	6.12 ± 1.27	7.08 ± 1.64 *	5.65 ± 1.01	5.82 ± 1.11	5.87 ± 1.76	5.97 ± 1.37
CREAT ** [µmol/L]	81.31 ± 24.6	65.99 ± 12.3	71.98 ± 7.45	72.87 ± 5.44	80.79 ± 9.25	71.24 ± 4.67	79.86 ± 20.1	74.6 ± 14.77	78.9 ± 16.44	67.84 ± 10.4	83.52 ± 20.7	72.78 ± 10.1
CRP [mg/L]	1.71 (IQR)	2.68 (IQR)	2.86 (IQR)	2.19 (IQR)	2.13 (IQR)	3.15 (IQR)	1.66 (IQR)	2.75 (IQR)	2.63 (IQR)	1.54 (IQR)	2.80 (IQR)	1.50 (IQR)
FBG [g/L]	3.34 ± 1.10	3.14 ± 0.52	3.06 ± 0.79	3.65 ± 0.11 **	3.1 ± 0.65	3.31 ± 0.28	3.3 ± 0.50	3.44 ± 0.70	3.27 ± 0.76	3.94 ± 0.62 **	3.49 ± 0.69	3.41 ± 0.51
Leu [10 × 9/L]	7.23 ± 2.07	6.87 ± 2.72	7.06 ± 1.56	7.73 ± 0.24	6.47 ± 1.83	6.48 ± 1.21	7.4 ± 2.05	7.68 ± 1.88	7.2 ± 1.30	9.81 ± 2.62 *	6.58 ± 1.36	7.68 ± 1.54
LCN-2 [pg/mL]	3593.6 ± 555	3175 ± 705.7	-	2948.84 ± 812	3245.8 ± 842.6	3067.6 ± 655.8	3558.4 ± 70.9	4207.7 ± 764	3652.9 ± 731.0	3418.3 ± 55.7	3681.4 ± 508.1	3277.2 ± 765.1
GDF-15 [pg/mL]	1312 ± 386.1	1559.9 ± 669	1365.8 ± 569	1608.9 ± 611	1462.9 ± 135.7	1479.9 ± 125.1	1540.2 ± 291.9	1373.8 ± 536	1652.40 ± 391.73 **	1479.73 ± 154.51	1492.72 ± 358.21	1538.95 ± 203.03
IL-6 [pg/mL]	5.03 (IQR)	6.16 (IQR)	5.52 (IQR)	9.25 (IQR)	1.65 * (IQR)	0.48 (IQR)	2.45 (IQR)	3.81 (IQR)	4.06 (IQR)	1.71 (IQR)	1.46 ** (IQR)	1.59 (IQR)
TIM-3 [pg/mL]	79.43(IQR)	129.43 (IQR)	120.86 (IQR)	80.14 (IQR)	142.29 (IQR)	225.14 (IQR)	139.43(IQR)	199.43(IQR)	130.86 (IQR)	99.43 (IQR)	125.14 (IQR)	219.43 * (IQR)

* BP—blood pressure; CREAT **—Creatinine; CRP—(C-reactive protein); FBG—fibrinogen; Leu-leukocytes; LCN-2—lipocalin-2; IQR—interquartile range.

**Table 3:** Correlation analysis in the group of patients divided according to the findings on coronary angiography.

	Age	Gender 0-M 1-F	BP	Urea [mmol/L]	CREAT [µmol/L]	CRP [mg/L]	FBG [g/L]	Leu [10 × 9/L]	LCN-2 pg/mL	GDF-15 pg/mL	IL-6 pg/mL	TIM-3 pg/mL
Pearson Correlation (2-tailed)	0.044	−0.225 **	−0.186 *	0.044	0.127	−0.023	0.100	0.049	0.125	0.120	−0.033	0.055
*p*-value	0.618	0.010	0.035	0.619	0.151	0.796	0.256	0.585	0.273	0.176	0.709	0.546
N	130	130	130	130	130	130	130	129	79	128	128	125

* = *p* ≤ 0.05, ** = *p* ≤ 0.01.

**Table 4:** Frequency of findings on coronary angiography in individual sexes.

Men			Women		
Finding	Frequency	Percentage	Finding	Frequency	Percentage
RADS 0	18	21.2	RADS 0	16	35.6
RADS 1	5	5.9	RADS 1	4	8.9
RADS 2	6	7.1	RADS 2	5	11.1
RADS 3	14	16.5	RADS 3	10	22.2
RADS 4	27	31.8	RADS 4	5	11.1
RADS 5	15	17.6	RADS 5	5	11.1
Total	85	100.0	Total	45	100.0

**Table 5:** Correlation analysis between findings and blood pressure.

Finding	N	Mean	SD	Variance	Range	Min.	Max.
RADS 0	34	146.618	15.3603	235.940	70.0	110.0	180.0
RADS 1	9	141.111	13.4112	179.861	45.0	125.0	170.0
RADS 2	11	146.364	21.6900	470.455	85.0	115.0	200.0
RADS 3	24	136.875	16.6689	277.853	65.0	100.0	165.0
RADS 4	32	138.281	18.2880	334.451	80.0	100.0	180.0
RADS 5	20	139.500	17.370	318.158	75.0	105.0	180.0

**Table 6:** Pearson correlation analysis in groups of patients divided according to findings and their differences between the sexes.

Men				Women		
	Pearson Correlation (2-Tailed)	*p*-Value	N	Pearson Correlation (2-Tailed)	*p*-Value	N
age [years]	−0.007	0.949	85	0.256	0.090	45
O_DM	0.156	0.155	85	0.090	0.555	45
blood pressure	−0.238 *	0.028	85	−0.067	0.663	45
Urea [mmol/L]	−0.013	0.904	85	0.174	0.253	45
CREAT [µmol/L]	0.029	0.795	85	0.201	0.187	45
CRP [mg/L]	0.030	0.784	85	−0.152	0.318	45
FBG [g/L]	0.051	0.644	85	0.294	0.050	45
Leu [10 × 9/L]	−0.060	0.588	84	0.262	0.082	45
lipocalin 2 [pg/mL]	0.099	0.483	52	0.135	0.504	27
GDF-15 [pg/mL]	0.271 *	0.013	83	−0.081	0.595	45
IL-6 [pg/mL]	0.010	0.928	84	−0.109	0.483	44
TIM-3 [pg/mL]	0.017	0.877	82	0.218	0.160	43

* = *p* ≤ 0.05.

**Table 7:** Descriptive statistics of GDF-15 expression in male patients divided into groups according to findings on coronary angiography.

Finding	N	Mean	Median	SD	Variance	Range	Min.	Max.
RADS 0	17.00	1312.14	1278.00	386.15	149,114.67	1626.05	694.65	2320.70
RADS 1	5.00	1365.84	1580.36	569.27	324,070.84	1292.38	751.09	2043.47
RADS 2	6.00	1462.93	1491.41	135.78	18,435.04	314.40	1292.28	1606.68
RADS 3	13.00	1540.23	1533.77	291.90	85,203.77	1249.67	1052.25	2301.92
RADS 4	27.00	1652.40	1643.20	381.73	145,720.32	1428.50	951.61	2380.11
RADS 5	15.00	1492.72	1427.98	358.21	128,311.75	1433.74	1111.36	2545.10

**Table 8:** Descriptive statistics of fibrinogen expression in female patients divided into groups according to findings on coronary angiography.

FBG	N	Mean	Median	SD	Variance	Range	Min.	Max.
RADS 0	16	3.14	3.10	0.52	0.27	1.70	2.30	4.00
RADS 1	4	3.65	3.62	0.11	0.01	0.25	3.56	3.81
RADS 2	5	3.31	3.25	0.28	0.08	0.74	3.00	3.74
RADS 3	10	3.44	3.47	0.70	0.49	2.61	2.39	5.00
RADS 4	5	3.94	3.70	0.62	0.39	1.55	3.37	4.92
RADS 5	5	3.41	3.47	0.51	0.26	1.24	2.87	4.11

## Data Availability

Raw data used during the current study are available from the corresponding author on reasonable request for non-commercial use.
